# Supplementary material for: Toxicity of Zn-Fe Layered Double Hydroxide to Different Organisms in the Aquatic Environment
Source: Molecules. 2021 Jan 13;26(2):395. doi: 10.3390/molecules26020395 (PMC7828569; doi:10.3390/molecules26020395)
Supplement: Supplementary file 1 [file molecules-26-00395-s001.pdf]

Supplementary Data

Toxicity of Zn-Fe Layered Double Hydroxide to Different Organisms in the Aquatic Environment

Olga Koba-Ucun [1], Tuğba Ölmez Hanci [1], Idil Arslan-Alaton [1,*], Samira Arefi-Oskoui [2], Alireza Khataee [2,3,*], Mehmet Kobya [3,4] and Yasin Orooji [5]

[1] School of Civil Engineering, Department of Environmental Engineering, Istanbul Technical University, 34469 Maslak, Istanbul, Turkey; kobaucun@itu.edu.tr (O.K.-U.); tolmez@itu.edu.tr (T.Ö.H.)
[2] Research Laboratory of Advanced Water and Wastewater Treatment Processes, Department of Applied Chemistry, Faculty of Chemistry, University of Tabriz, 51666-16471 Tabriz, Iran; s.arefi@tabrizu.ac.ir
[3] Department of Environmental Engineering, Gebze Technical University, 41400 Kocaeli, Turkey; kobya@gtu.edu.tr
[4] Department of Environmental Engineering, Kyrgyz-Turkish Manas University, 720038 Bishkek, Kyrgyzstan
[5] College of Materials Science and Engineering, Nanjing Forestry University, Nanjing 210037, China; yasin@njfu.edu.cn
* Correspondence: arslanid@itu.edu.tr (I.A.-A.); akhataee@gtu.edu.tr (A.K.)

Table S1. Summary of toxicity test procedures used in this study.

Test Organism	Taxonomy/ Trophic Level	Concentration Range (g/L)	Exposure Time	EC_{50} (g/L)	Toxicity Endpoint	Test Protocol
V. fischeri	Photobacteria/ decomposer	0.05-2.00	15 min	0.31±0.03[a]<br>1.20±0.12[b]	Bioluminescence inhibition	ISO 11348-3
			30 min	0.11±0.01[a]<br>0.32±0.03[b]		
P. subcapitata	Freshwater algae/ producer	0.10-2.00	72 h	< 0.1[c]	Growth inhibition	ISO 8692
D. magna	Cladoceran/ consumer	0.05-2.00	24 h<br>48 h	0.17±0.02<br>0.10±0.01	Death or immobilization	OECD 202
S. polyrhiza	Aquatic plant/ producer	0.075-2.00	72 h	0.81±0.19	Growth inhibition	ISO 20227

[a] Preparation time for the catalyst was 1 h; [b] Preparation time for the catalyst was 3 h; [c] Complete inhibition was observed at all tested catalyst concentrations.

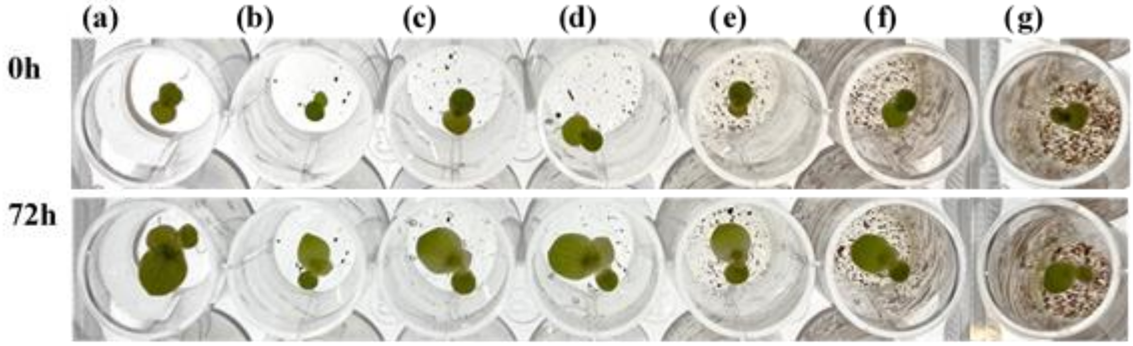

Figure 1. Changes in the fronds of *S. polyrhiza* exposed to the different concentrations of Zn-Fe LDH. The figure shows fronds for test control (a), 0.05 g/L (b), 0.075 g/L (c), 0.10 g/L (d), 0.50 g/L (e), 1.00 g/L (f), and 2.00 g/L (g) Zn-Fe LDH exposure.
